# A highly sensitive safrole sensor based on polyvinyl acetate (PVAc) nanofiber-coated QCM

**DOI:** 10.1038/s41598-019-51851-0

**Published:** 2019-10-28

**Authors:** Kuwat Triyana, Aditya Rianjanu, Doni Bowo Nugroho, Ahmad Hasan As’ari, Ahmad Kusumaatmaja, Roto Roto, Risa Suryana, Hutomo Suryo Wasisto

**Affiliations:** 1 Department of Physics, Faculty of Mathematics and Natural Sciences, Universitas Gadjah Mada, Yogyakarta, 55281 Indonesia; 2 Institute of Halal Industry and System (IHIS), Universitas Gadjah Mada, Sekip Utara, Yogyakarta, 55281 Indonesia; 3 Department of Chemistry, Faculty of Mathematics and Natural Sciences, Universitas Gadjah Mada, Yogyakarta, 55281 Indonesia; 4 Department of Physics, Faculty of Mathematics and Natural Sciences, Sebelas Maret University, Surakarta, 57126 Indonesia; 5 Institute of Semiconductor Technology (IHT) and Laboratory for Emerging Nanometrology (LENA), Technische Universität Braunschweig, Braunschweig, 38106 Germany

**Keywords:** Sensors, Analytical chemistry

## Abstract

A novel, highly sensitive and selective safrole sensor has been developed using quartz crystal microbalance (QCM) coated with polyvinyl acetate (PVAc) nanofibers. The nanofibers were collected on the QCM sensing surface using an electrospinning method with an average diameter ranging from 612 nm to 698 nm and relatively high *Q*–factors (rigid coating). Scanning electron microscopy (SEM) and atomic force microscopy (AFM) were used to analyze the PVAc nanofiber surface morphology, confirming its high surface area and roughness, which are beneficial in improving the sensor sensitivity compared to its thin-film counterpart. The as-spun PVAc nanofiber sensor could demonstrate a safrole limit of detection (LOD) of down to 0.7 ppm with a response time of 171 s and a sensitivity of 1.866 Hz/ppm. It also showed good reproducibility, rapid response time, and excellent recovery. Moreover, cross-interference of the QCM sensor response to non-target gases was investigated, yielding very low cross-sensitivity and high selectivity of the safrole sensor. Owing to its high robustness and low fabrication cost, this proposed sensing device is expected to be a promising alternative to classical instrumental analytical methods for monitoring safrole-based drug precursors.

## Introduction

Electrospinning is a simple and versatile technique that can create a micron–thick film containing sub-micron scale nanofibers. This technique is used to fabricate nanofibers for various applications ranging from membrane filtration to tissue engineering, and more recently, electrochemical applications^1–6^. The development of nanostructured materials, especially nanofiber as a sensing material, has been accelerated over the past decades^7^. Electrospun nanofiber film offers fibers with very small diameters and large surface areas per unit mass, which are advantageous for gas sensing^8^. The high specific surface area and porosity of electrospun nanofibers have sparked an increasing interest in their use as ultrasensitive gas sensors^9–12^. Thus, they have recently been used as active sensing layers and integrated onto various gas sensor platforms including resistive^13^, photoelectric^14^, amperometric^15^, optical^16^, and acoustic wave^17,18^. Among these devices, gravimetric sensors based on quartz crystal microbalance (QCM) have been preferred to be used for vapor detection because of their high sensitivity, fast response, inexpensive production, real-time measurement capability, and simple integration with other electronic components^19–24^. The QCM is basically a piezoelectric sensor platform employing acoustic-electric effects that can measure mass resolution down to the picogram level^25^. By modifying the QCM chip sensing area with appropriate sensing layers (e.g., polymers^26–29^, metal oxides^30,31^, and some functional materials^32,33^), specific analytes that are additionally deposited on its surface can induce a frequency shift, which can subsequently be translated into detected analyte mass according to the Sauerbrey equations^34^.

Safrole is a pale–yellow oil that is normally used as a precursor in the production of the amphetamine-type stimulus drug 3,4–methylenedioxyamphetamine (MDMA, “ecstasy”). Several studies have highlighted the need for the development of a better technique for the detection of drug–synthesis reactants (i.e., benzodioxol, benzyl methyl ketone (BMK), and safrole)^35–38^. Conventional analytical methods (e.g., liquid chromatography (LC), gas chromatography (GC), and gas chromatography coupled with mass spectrometry (GC-MS)^39,40^) are often employed for forensic safrole testing. However, these techniques are costly, not portable, and challenging to be operated in real-time. Therefore, the development of a rapid, reliable, low-cost, and real-time safrole sensor is needed. In our previously reported studies^37,38^, although nanofiber-based QCM safrole sensors had been developed and tested, their performance was still unsatisfactory in terms of sensitivity and selectivity, due to poor intermolecular interaction between safrole molecules and sensing layers (i.e., polyacrylonitrile (PAN) nanofiber). Therefore, polyvinyl acetate (PVAc)-based nanofiber membrane was developed and employed in this work to overcome this issue. This hydrophobic polymer could be easily electrospun on the active layer of QCM. The hydrophobicity of PVAc is essential since the response of the QCM vapor sensor is heavily interfered by water vapor. For the sensing mechanism, the oxygen molecules in PVAc structure will act as a Lewis base that can interact with safrole (Lewis acid). A Lewis base is an electron pair donor, while a Lewis acid is an electron pair acceptor^41^. They can react with each other forming a covalent bond, where both electrons are provided by the Lewis base. In case of their molecular orbitals, Lewis acids and bases have an unoccupied low-energy atomic states and occupied relatively high energy atomic states, respectively. A lone electron pair (i.e., a pair of valence electrons that are not shared with another atom in a covalent bond) in oxygen sites from each PVAc unit may interact strongly with two protons located between two oxygen atoms of safrole. Furthermore, to demonstrate its feasibility as a practical sensor in a real field, cross-sensitivity measurements toward other non-target gases were also conducted and evaluated.

## Results and Discussion

### PVAc membrane analysis

Figure 3 displays the SEM images of both PVAc thin-film (NF 0) and PVAc nanofibers (NF 1, NF 2, and NF 3). Continuous nanofiber structures with a smooth surface are clearly seen from the inset images of Fig. 3(b–d). The mean diameters of the nanofiber samples are listed in Table 1, revealing similar average diameters ranging from 612 nm to 698 nm. Meanwhile, the morphology of PVAc thin-film produced by a spin coating process revealed a smooth surface with several pores (see Fig. 3(a)). Both types of structures presented relatively homogenous features in their physical morphology. Furthermore, they also possessed 3D pore architectures that led to easy accessibility and fast exposure to the gases, rendering the PVAc nanostructured membrane a potential candidate for application in routine vapor detection. The 3D porous structure increased the sensor surface area and enhanced the contact area between safrole molecules and sensing sites. Thus, it could improve the sensing performances. Table 1 also shows the sensing layer thickness *h*_sensor_, which is calculated using Eq. (1)

1 $${h}_{sensor}=\frac{{m}_{sensor}}{{\rho }_{sensor}\times {A}_{QCM}}$$

where ${m}_{sensor}$, ${\rho }_{sensor}$, and ${A}_{QCM}$ are the deposited sensor mass (see Table 1), the density of PVAc (1.19 g/cm^3^), and the surface area of QCM electrodes (0.283 cm^2^), respectively.

**Figure 1:**
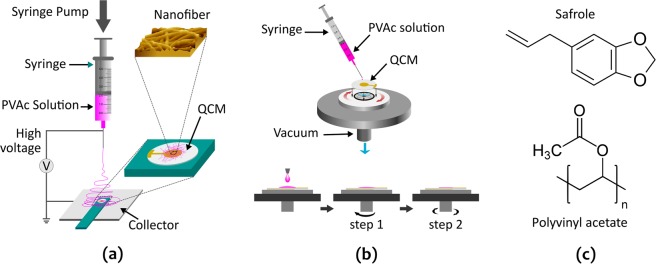
Schematic illustration of (**a**) electrospinning setup, (**b**) a spin-coating process, and (**c**) chemical structure of polyvinyl acetate (PVAc) and safrole.

**Figure 2:**
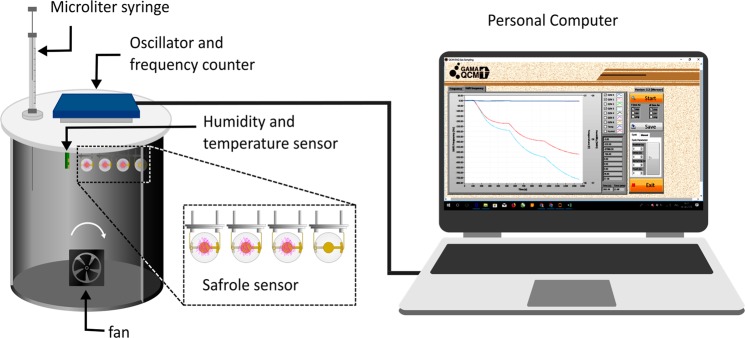
Measurement setup of the quartz crystal microbalance system for gas or vapor sensing.

**Figure 3:**
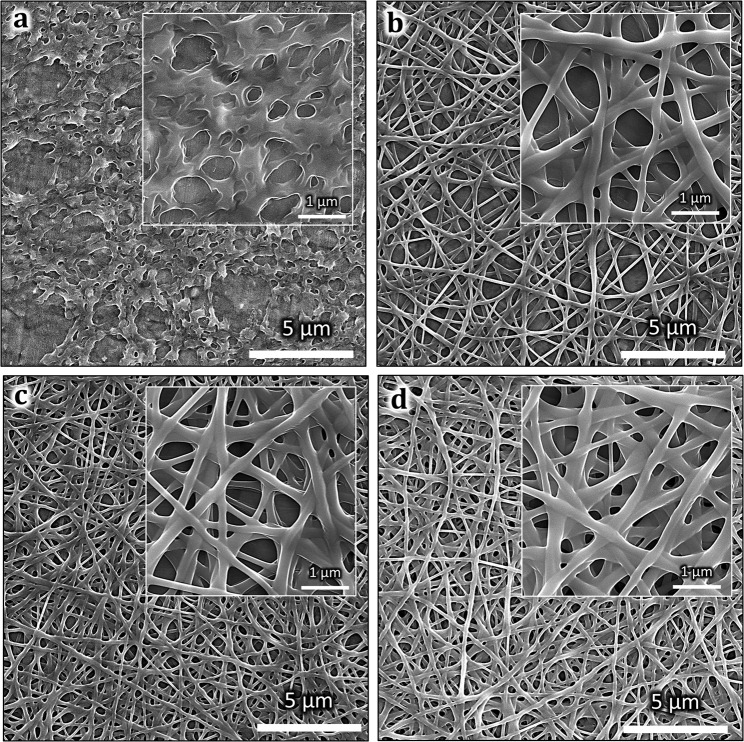
SEM images of (**a**) PVAc thin-film (NF 0), (**b**) PVAc nanofiber (NF 1), (**c**) NF 2, (**d**) NF 3 (inset figure is the magnified view of the structures).

**Table 1:** Parameters of developed PVAc sensors (i.e., NF 0, NF 1, NF 2, and NF 3) based on different membrane structures (i.e., thin-film and nanofibers).

Sample	NF 0	NF 1	NF 2	NF 3
Membrane form	Thin-film	Nanofiber	Nanofiber	Nanofiber
Deposition time (s)	30	5	15	25
Diameter (nm)	—	631 ± 85	612 ± 98	698 ± 70
Frequency shift (kHz)	16.3	2.0	16.2	21.0
Deposited mass (µg)	20.4	2.5	20.2	26.2
Calculated thickness (nm)	606	74	599	778

The density of PVAc and the surface area of QCM electrode are 1.19 g/cm^3^ and 0.283 cm^2^, respectively.

**Figure 4:**
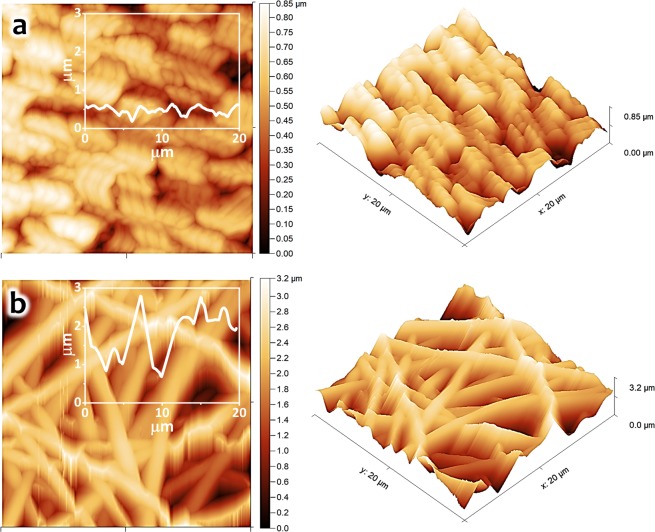
Top-view and cross-sectional AFM images of the PVAc nanostructures: (**a**) NF 0 and (**b**) NF 2.

Figure 4(a,b) show the AFM analyses of deposited PVAc thin film and nanofibers. In the case of the PVAc thin-film (NF 0), a relatively homogeneous surface with several porous structures was obtained. The root mean square roughness (R_RMS_) of the PVAc thin-film sample was found at about 140 nm, with a 415 nm^2^ surface area (Fig. 4(a)). To the contrary, the PVAc nanofiber sample (NF 2) was clearly found to be rougher than its thin-film surface, which was confirmed by its R_RMS_ of about 520 nm, with a 572 nm^2^ surface area (Fig. 4(b)). It should be mentioned that both R_RMS_ values obtained for thin-film and nanofiber samples were determined on the basis of the 20 × 20 μm^2^ of the AFM images. Besides, because of their similar morphologies shown in Fig. 3(b–d), other two nanofiber samples (NF 1 and NF 3) are expected to have similar roughness and porosity characteristics to sample NF 2.

**Figure 5:**
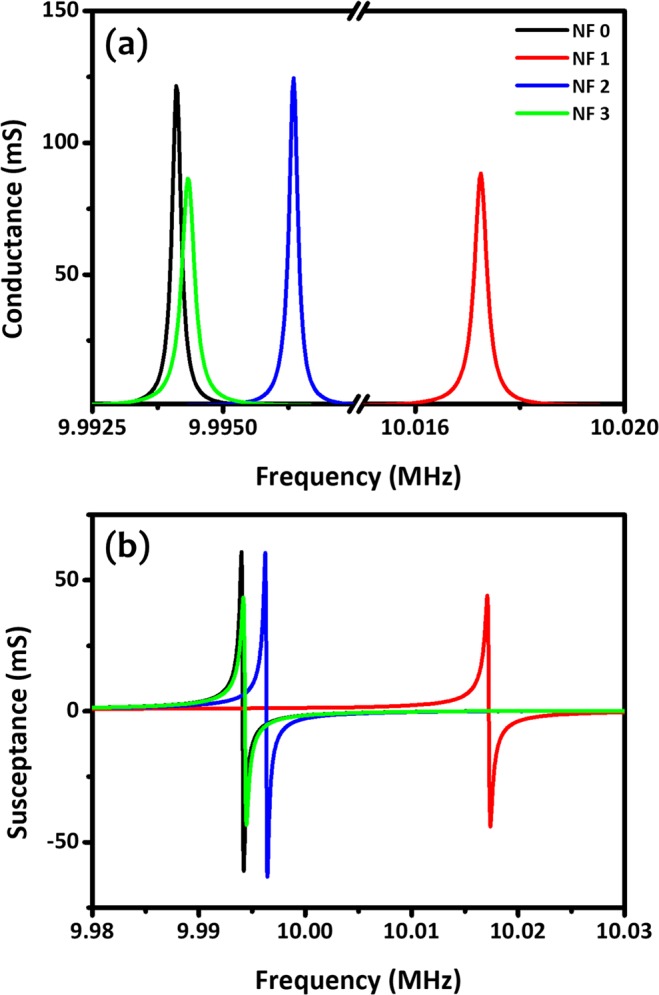
Frequency characteristics of all sensors (NF 0–NF 3) measured in (**a**) conductance and (**b**) susceptance modes.

The conductance and susceptance spectra were measured to analyze the resonant characteristics of the QCM-based PVAc sensor prior to safrole exposure as shown in Fig. 5(a,b). Accordingly, quality factor (Q–factor) of the resonator could be extracted as the ratio of the peak frequency to the half bandwidth in the conductance spectrum. It is well-known from micro-/nanoelectromechanical systems (MEMS/NEMS), that higher Q-factor is preferable for sensors to have better stability, smaller frequency noise, and less energy loss^31,42^. The measured Q–factors of the QCM-based PVAc sensors in normal ambient air were 47818, 32951, 48059, and 33093 for NF 0, NF 1, NF 2, and NF 3, respectively. In comparison with other typical MEMS/NEMS-based resonators made of piezoresistive silicon cantilevers^43–45^, electrothermal silicon cantilevers^46–48^, and vertical silicon nanowires^49–51^, the piezoelectricity QCM sensors (quartz) exhibited superiority in terms of Q–factor, even after being functionalized with PVAc thin-films or nanofibers. This demonstrated that both polymer deposition techniques used in this work (i.e., spin coating and electrospinning) could offer a solution for creating stable hybrid polymer/semiconductor MEMS-based devices with low-frequency noise. It should be noted that for such silicon microcantilever structures coated with photoresist in a drop-casting method, their Q–factors may deteriorate by one order of magnitude^52^.

### Safrole exposure assessment

**Figure 6:**
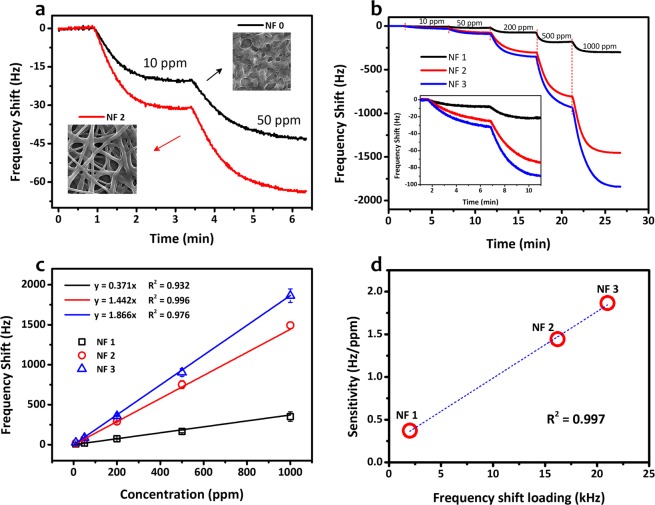
QCM-based PVAc safrole sensor characteristics. (**a**) Frequency responses of PVAc thin-film (NF 0) and PVAc nanofiber (NF 2). (**b**) Dynamic responses of PVAc nanofiber sensor for increasing safrole concentration (inset: zoom at low concentration). (**c**) Frequency shift of QCM PVAc safrole sensor in different safrole concentrations. (**d**) Sensitivity of QCM PVAc nanofiber sensor for increasing loading depositions.

Figure 6(a) shows the measured dynamic responses of QCM sensors coated with PVAc thin-film (NF 0) and PVAc nanofiber (NF 2) under exposure to safrole vapors. To directly investigate the influence of surface-to-volume enhancement of the nanofibers, both samples were previously treated with similar loading depositions (i.e., ~16 kHz). During the gas sensing test, the safrole concentration was initially set at 10 ppm and subsequently increased to 50 ppm. As expected, both sensors (NF 0 and NF 2) experienced frequency shifts toward lower values. However, from the sensor responses, it was clearly shown that the nanofiber structure (NF 2) exhibits larger frequency shifts (i.e., around 1.5 times) compared to its thin-film counterpart (NF 0), even though PVAc deposition masses for both devices were kept at a similar level. At 50 ppm safrole concentration, the frequencies of NF 0 and NF 2 samples dropped by 42 Hz and 65 Hz, respectively, from their initial value, where the gas had not been injected into the sealed chamber. This result has confirmed the importance of having larger surface area and porous surface structures made as nanofibers compared with ordinary thin film^53^. This argument for the response difference between these two sensors has been supported by the SEM (Fig. 3(a,c)) and AFM images (Fig. 4(a,b)). The SEM images indicate that the thin-film sample had a relatively smooth surface with a few pores, while the nanofiber sample possessed more membrane pores (space between the nanofibers). Moreover, the AFM images show that the nanofiber structure had a rougher surface and higher surface area compared to the thin film.

To investigate the influence of loading deposition on QCM-based PVAc nanofiber sensor performance, the electrospinning parameters were varied as listed in Table 1. The loading deposition was increased from NF 1 to NF 3. Figure 6(b) depicts the dynamic responses of three sensors with different nanofiber structures under exposure to increasing safrole concentrations from 10 to 1000 ppm. It shows that the response of the PVAc nanofiber sensors could be enhanced by increasing the loading depositions. For instance, at 50 ppm safrole concentration, the responses (changes of resonance frequency) of the sensors were measured to be 18 Hz, 71 Hz, and 85 Hz for NF 1, NF 2, and NF 3 sensors, respectively. Moreover, by increasing the safrole concentration up to 1000 ppm, a larger frequency change in the QCM sensors was obtained, reaching up to almost 2 kHz (Fig. 6(c)). The sensitivity, which is defined as the slope of the linear correlation between the safrole concentration and its frequency change, also increased from NF 1 to NF 3 (i.e., *S*_NF1_ = 0.37 Hz/ppm, *S*_NF2_ = 1.44 Hz/ppm, and *S*_NF3_ = 1.86 Hz/ppm). A good linear correlation between loading deposition (frequency shift after coating) and sensor sensitivity is shown in Fig. 6(d) with a correlation coefficient (*R*^2^) of 0.997. The increase in loading depositions indicates that more PVAc molecules were successfully deposited on the QCM chips. The feasibility of intermolecular interaction between the active layer and the analyte was therefore enhanced, resulting in higher frequency shifts.

### Proposed sensing mechanism

**Figure 7:**
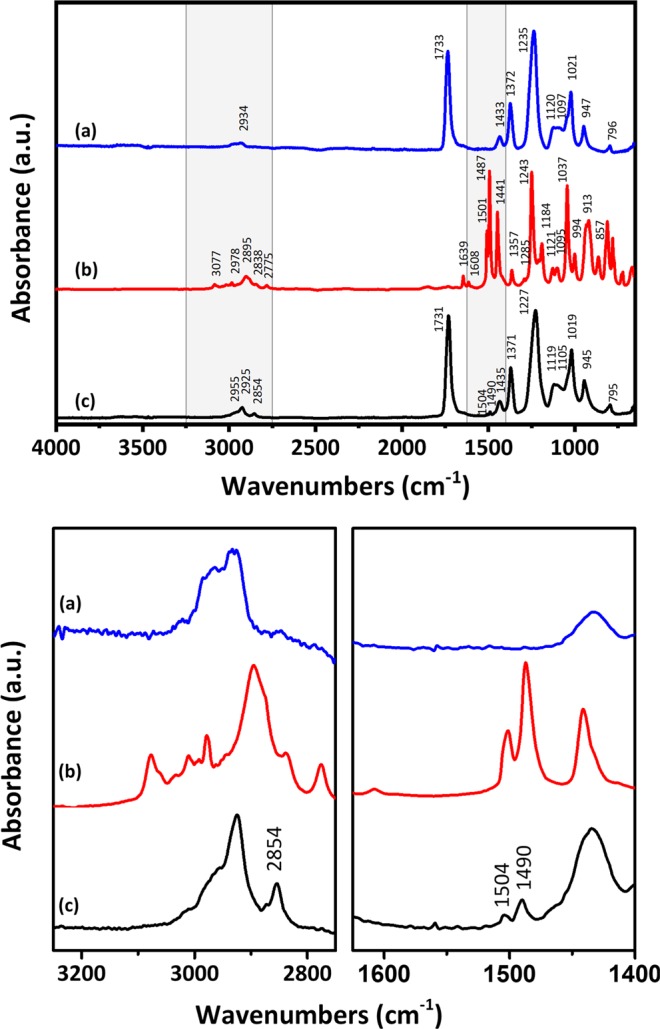
FTIR spectra of (**a**) PVAc nanofiber, (**b**) safrole, and (**c)** PVAc exposed to safrole vapor.

Physisorption or physical adsorption between safrole gas and PVAc can be an important adsorption mechanism toward an understanding of the sensing behavior of this developed hybrid PVAc/QCM device. From FTIR characterization, we could confirm that there was no chemical interaction between PVAc nanofiber and safrole molecules. Figure 7(a–c) show the IR spectra of PVAc, safrole, and PVAc exposed to safrole, respectively. PVAc nanofiber (Fig. 7(a)) had an absorbance at the peak of 1731–1733 cm^−1^, which was attributed to an acetate ester carbonyl group (C=O; C–O). The other peaks could be found at 1433–1435 cm^−1^ (C–H), 1371–1372 cm^−1^ (C–H), 1227–1235 cm^−1^ (C–O), and 1019–1021 cm^−1^ (C–O)^54^. IR spectrum of safrole is shown in Fig. 7(b), with peaks at 1501 cm^−1^, 1490 cm^−1^, and 1441 cm^−1^, that are related to stretching of the carbon double bond (C=C), while an additional peak at 3077 cm^−1^ can be attributed to stretching of the hydrocarbon (C–H), which is an aromatic group. Furthermore, safrole has an aldehyde group that is depicted by peaks at 2775 cm^−1^, 2838 cm^−1^, 2895 cm^−1^, and 2978 cm^−1^. Figure 7(c) shows the peak characteristics of both PVAc nanofiber and safrole. The peaks observed in the PVAc nanofiber exposed to safrole FTIR spectra were close to those in the spectrum of PVAc. The peaks detected at 1504 cm^−1^ dan 1490 cm^−1^ (C=C) were attributed to the aromatic ring, whereas peaks at 2955 cm^−1^ and 2854 cm^−1^ (C–H) corresponded to the aldehyde group of the safrole. These results confirmed that no chemical reaction occurred when PVAc nanofiber was exposed to safrole. The only possible interaction was intermolecular interaction, which cannot be detected using the FTIR method.

**Figure 8:**
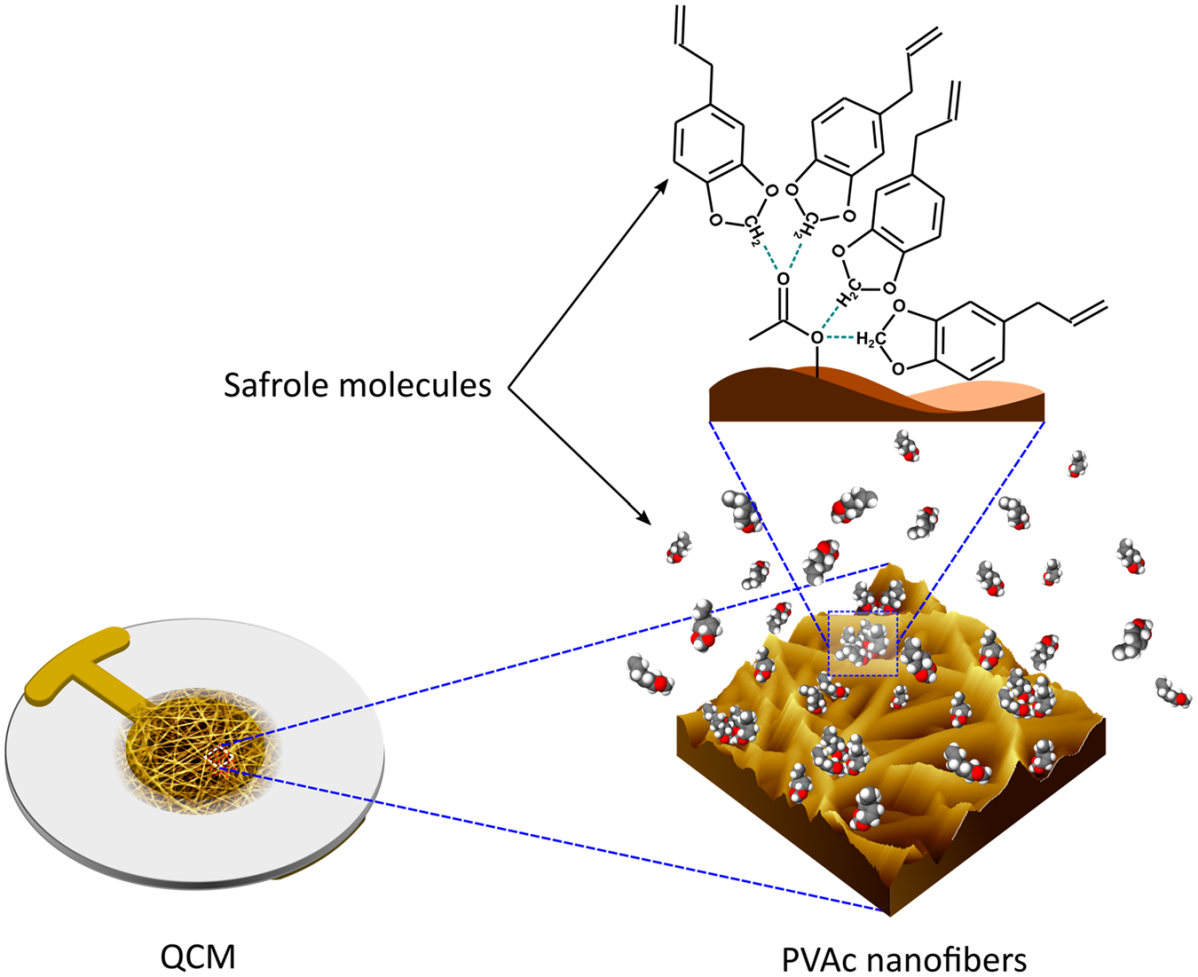
Schematic representation of the proposed interaction mechanism between the PVAc nanofiber and safrole molecules.

There may be two scenarios by which safrole is received by the sensor as illustrated in Fig. 8. First, the carbonyl group, an ester of PVAc, has an O atom with a higher electronegativity than the C atom of safrole. In this situation, the O atom has a partial negative charge (δ^−^) and the C atom has a partial positive charge (δ^+^). Consequently, dipole-dipole interactions can occur. Furthermore, one O atom of PVAc monomer can interact with the carbon atoms of two safrole molecules, so that one monomer of PVAc can interact with four safrole molecules. Because of these phenomena, PVAc can interact more readily than PAN, used in former works^37^. Secondly, the molecular structure may influence the interaction and cause different sensor sensitivities to the analyte. PVAc is a polymer that has a polar structure, so that interactions will be stronger with analyte molecules that also have a polar structure^55^. Each analyte has a unique molecular structure affecting its polarity.

### Other sensor characteristics

The LOD and the limit of quantification (LOQ) are determined based on the standard deviation and the slope of the plot in Fig. 6(c)^56^. The LOD and LOQ are expressed as 3.3 *σ/S* and 10 *σ/S*, respectively, where *σ* is the standard deviation of the blank air measurement (i.e., 0.37 Hz for NF 3) and *S* is the sensor sensitivity (i.e., 1.866 Hz/ppm for NF 3). Thus, the LOD and LOQ of QCM modified with PVAc nanofiber (NF 3) could be calculated to be 0.7 ppm and 2 ppm, respectively.

Compared to other materials, the PVAc nanofiber sensors produced by electrospinning exhibited the highest sensitivity. While integrating PAN nanofiber on QCM devices only resulted in sensor sensitivities of 0.035 Hz/ppm^37^, using PVAc nanofiber (NF 3) could deliver a sensitivity of up to 1.86 Hz/ppm, which demonstrated a remarkable increase of ~53 times. The explanation of this phenomenon has been discussed previously in the proposed sensing mechanism section.

**Figure 9:**
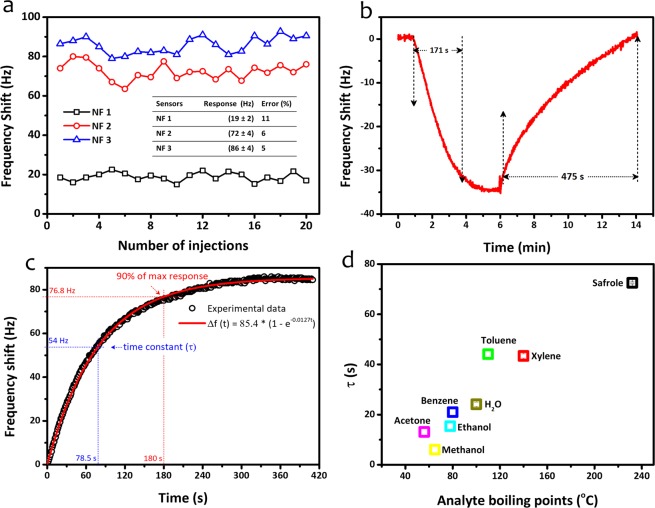
(**a**) Reproducibility, (**b**) response–recovery, (**c**) response time of NF 2 samples exposed to 50 ppm of safrole, and (**d**) time constant of the NF 2 sensor exposed to various analytes corresponding to its boiling points.

To investigate their sensing reproducibility, the PVAc nanofiber sensors were exposed to safrole vapors at a fixed concentration of 50 ppm and multiple gas injections (up to 20 times). Figure 9(a) shows the measured signals of three different QCM-based PVAc sensors during the reproducibility test. The standard deviations were found to be 11, 6, and 5% for the response values of NF 1, NF 2, and NF 3, respectively. The slight fluctuation in frequency shift suggests that all QCM chips exhibited high sensing reproducibility.

The full sensor response and recovery characteristics are important parameters in the performance of gas sensors. Thus, to investigate them in the PVAc sensors, we modified the gas sensing configuration. The response-recovery investigation was performed by modifying the sensor chamber in accordance with previously reported setup^9,24^. The safrole concentration was predetermined at 50 ppm. The response and recovery behaviors of the NF 2 sensor are depicted in Fig. 9(b), where full recovery could be obtained by the sensor after being exposed to safrole. Moreover, the response and recovery times for the NF 2 sensor were measured to be 171 and 475 s, respectively. It should be noted that the sensor response or recovery time is defined as the time required for the sensor to reach 90% of its maximum value (t_90_) during response or recovery activity. In comparison to typical conductometric metal oxide gas sensors^57,58^, which need longer response/recovery times (10–120 min), our PVAc-coated QCM sensors could, apparently, react faster to exposing gas.

Figure 9(c) shows the response of the NF 3 sensor to safrole vapor at 50 ppm, which fits the first-order instrument response. The time constant (τ) was found to be 78 s for this device toward safrole. For other analytes, the sensor time constant also differed, depending on the interaction between sensor and measured gas molecules. Regarding sensing, we believe that the properties of the analyte (i.e., boiling points) influenced the response times of the sensor. Figure 9(d) shows the correlation graph between the analyte boiling points and the relaxation time of the QCM-based PVAc sensor. The results indicate that the relaxation time increases linearly with the rise of the analyte boiling point for most of the analytes. The anomaly appears for the methanol analyte, for which factors other than analyte boiling point may have influenced the relaxation time. Thus, in-depth investigation into this matter will be necessary for our next study. Moreover, the results presented here only apply when the sensor responses were measured using static batch method.

**Figure 10:**
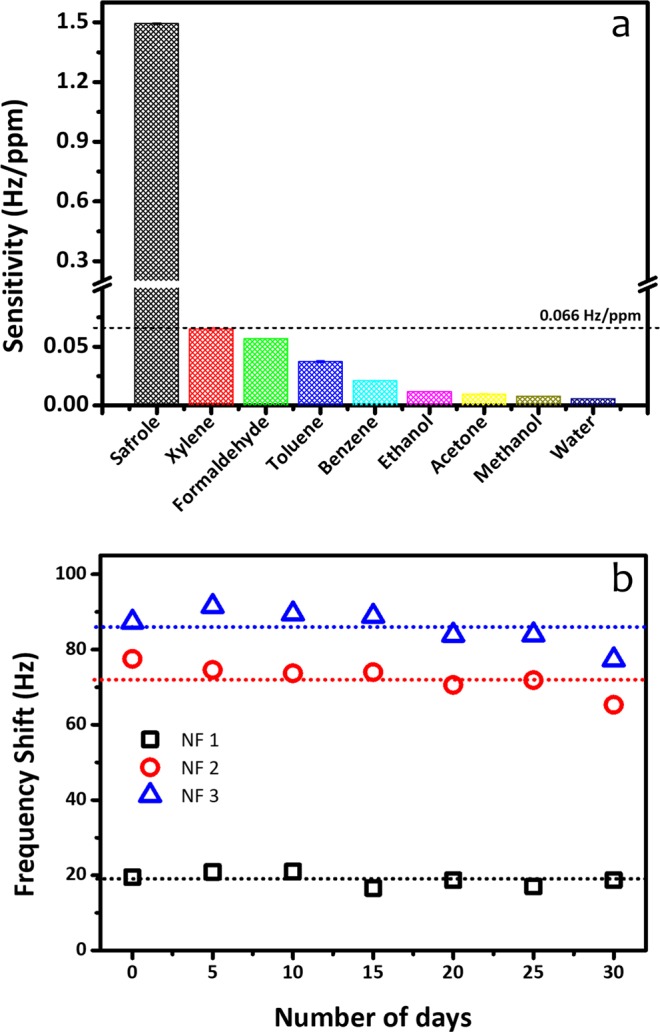
(**a**) Sensitivity comparison (NF 2 at 50 ppm safrole), and (**b**) long-term stability measurement of QCM PVAc nanofiber safrole sensor.

Apart from sensitivity and response/recovery time, reaction selectivity of the sensors plays an important role to examine the device practicability in the field. Therefore, cross-sensitivity experiments were conducted with various volatile organic compound (VOC) gases at 50 ppm concentration, which are usually present in ambient air (US EPA). As the sensor under test, we used the NF 2 sensor, whose results are shown in Fig. 10(a). Extremely high selectivity was observed for safrole gas, whose measured sensitivity is 1.44 Hz/ppm. Meanwhile, the PVAc nanofiber sensor showed a very low response toward other gases (e.g., xylene, formaldehyde, toluene, benzene, ethanol, acetone, methanol, and water), for which a sensitivity of below 0.065 Hz/ppm was normally obtained. Thus, it can be concluded that the PVAc nanofiber sensor (NF 2) showed an excellent selectivity to safrole vapor.

As sensor robustness is very important for real device applications, the PVAc safrole sensors were regularly tested with 50 ppm safrole vapor every 5 days over a 30 day period to evaluate their long-term stability. Figure 10(b) displays the responses (frequency shifts) as a function of time over 30 days. The average responses of the PVAc sensors (NF 1, NF 2, and NF 3) were measured to be 19, 72, and 86 Hz, with relative errors of 9, 4, and 5%, respectively. The sensor still displayed a good response with an almost constant value of resonance frequency after 30 days of gas sensing measurement, indicating that the employed QCM system is highly stable for long-term use.

## Conclusions

PVAc nanofibers have been successfully fabricated through a simple and facile electrospinning technique, in which their sensing characteristics toward safrole vapor have been investigated. The high surface area and porous structure of the nanofibers clearly influence the safrole sensor performance. Compared to the device coated with PVAc thin-film, the sensors functionalized with PVAc nanofibers exhibited an excellent enhancement of safrole detection. The response sensitivity of the PVAc nanofiber sensor is 1.5 times higher than that of PVAc thin-film sensor. The detection limit of the sensor could reach as low as 0.7 ppm, with up to 1.866 Hz/ppm sensitivity toward safrole. As–prepared sensors have shown good reproducibility, long-term stability, and high selectivity toward others analytes. All in all, the QCM sensing chip modified with electrospun polymers may offer a new strategy in real-time sensing of drug precursors.

## Materials and Method

### Materials

Polyvinyl acetate (PVAc) with a molecular weight of 500,000 g/mol was supplied by Sigma–Aldrich. N,N–dimethylformamide (DMF) for dissolving PVAc was purchased from Merck (Germany). Safrole analyte with 96% purity, was supplied by the Indonesian National Police, Jakarta, Indonesia. All chemicals were used as received without further purification. The AT–Cut QCM sensors with gold electrodes and 10 MHz base resonant frequency were purchased from OpenQCM, Novaetech. R&D, Napoli, Italy.

### Preparations of QCM PVAc nanofiber and thin-film

The PVAc solution was prepared using dimethylformamide (DMF) at a concentration of 15% (w/w) and subsequently electrospun on a QCM substrate at an applied voltage of 15 kV and a needle–to–collector distance of 15 cm. The electrospinning process (Fig. 1(a)) was carried out for 5, 15, and 25 seconds and denoted as NF 1, NF 2, and NF 3, respectively. After being collected on the chip, the as-spun nanofibers were then dried overnight prior to their use in sensor assessments.

To investigate the effect of enhanced surface area by nanofibers on QCM sensor performance, besides the electrospinning technique, a spin coating method was also utilized for producing a PVAc membrane with a different structure (i.e., thin-film) using Compact Spin Coater VTC–100 (see Fig. 1(b)). The PVAc thin-film sensor was fabricated using the same parameters as previously reported^24^. The two-step spin coating process was employed during the thin-film membrane fabrication to obtain a deposited film mass similar to that of the PVAc nanofiber sample created by electrospinning. Spinning speeds of 500 rpm and 5000 rpm were set during the first and second spin processes for 5 s and 30 s, respectively. The resulting PVAc thin-film membrane was then denoted as NF 0. The chemical structures of polyvinyl acetate (PVAc) and safrole are shown in Fig. 1(c).

### Sensor apparatus for safrole detection

A schematic diagram of the PVAc QCM testing system is shown in Fig. 2. The PVAc QCM sensor was installed inside a testing chamber (2 L) using similar environmental conditions as described in literature^24,28^. A ten microliter Hamilton syringe (Model 701 RN SYR) was used for analyte injection. The concentration of an injected analyte in the testing chamber was calculated according to Eq. (2)^53,59^:

2 $$C=(\frac{22.4\rho {V}_{s}T}{273MV})\times {10}^{3}$$

where *C* is the analyte concentration in ppm, $\rho$ is the density of liquid analyte in g/mL, ${V}_{s}$ is the volume of the analyte in µL, $T$ is the Kelvin temperature in the testing chamber, $M$ is the molecular weight of analyte in g/mol, and $V$ is the chamber volume in L. This equation assumes that the safrole is fully evaporated in the chamber after injection. The responses of the QCM sensors were tested by monitoring the change in the resonance frequency, as measured by a frequency counter. The change in the resonant frequency of the QCM sensor is related to the change in mass loading on the QCM sensor chip. The frequency change data were recorded with a PC equipped with the LabVIEW graphical programing language. To desorb the analyte vapor from the QCM sensing chips, dry ambient air was used during purging.

### Sample characterization

The nanofiber morphology of the samples was analyzed using scanning electron microscopy (SEM, JEOL JSM–6510). Their roughness was characterized using atomic force microscopy (AFM, XE–70, Park System Corp., South Korea). The conductance and susceptance of the QCM were measured using a vector network analyzer (Omicron–Lab Bode 100), while Fourier-transform infrared spectroscopy (FTIR, Thermo Nicolet iS10) was utilized to understand the chemical reactions between safrole gas and PVAc. The amount of mass deposited on the QCM chip can be calculated according to the Sauerbrey equation^34^, as expressed in Eq. (3):

3 $$\Delta f=-\frac{2{f}_{0}^{2}}{A\sqrt{{\rho }_{q}{\mu }_{q}}}\Delta m$$

where $\Delta f$ is the resonant frequency shift of QCM (Hz), ${f}_{0}$ is the base resonant frequency (10 MHz), $\Delta m$ is the mass change (g), $A$ is the electrode surface area (0.283 cm^2^), ${\mu }_{q}$ and ${\rho }_{q}$ are the shear modulus and density of quartz crystal (2.947 × 10^11^ g·cm^−1^·s^−2^ and 2.648 g·cm^−3^), respectively. The developed PVAc membranes (i.e., NF 0, NF 1, NF 2, and NF 3) with different fabrication parameters and masses on the surface of the QCM substrates are listed in Table 1. For cases of created nanofibers, regardless of their similar diameters, it is obvious that the NF 1 and NF 3 samples have the smallest and largest deposited masses, respectively, which differ by one order of magnitude, affecting the performance of the QCM sensors.
